# Effect of Freeze–Thaw Cycle Times on Basic Properties and Bond Performance with Steel Reinforcement of Tunnel Lining Concrete in the Tibetan Plateau

**DOI:** 10.3390/ma19101952

**Published:** 2026-05-09

**Authors:** Yamei Zang, Yulin Zhan, Dongchen Guo, Shixin Liang, Qi Zhao, Qinghua Tao, Hongfa Yu

**Affiliations:** 1 College of Engineering, Tibet University, Lhasa 850000, China; zangyamei@utibet.edu.cn (Y.Z.); 15518868233@163.com (Q.Z.); 2 School of Civil Engineering, Southwest Jiaotong University, Chengdu 610031, China; 3 Civil Aviation Engineering Quality Supervision Station, Beijing 100102, China; liangshixin@caac.gov.cn; 4 College of Civil Aviation, Nanjing University of Aeronautics and Astronautics, Nanjing 210016, China

**Keywords:** reinforced concrete, freeze–thaw cycles, bonding stress, maximum slip value, bond–slip curve

## Abstract

To investigate the bond performance between reinforcement and tunnel lining concrete under freeze–thaw cycles in plateau regions, pull-out tests were conducted on secondary-lining-reinforced concrete specimens subjected to different numbers of freeze–thaw cycles. The variations in the fundamental properties of the lining concrete, as well as the bond stress and maximum slip between the reinforcement and the concrete, were examined. The results indicate that, with an increasing number of freeze–thaw cycles, the mass of the lining concrete first increases and then decreases, while the compressive strength and splitting strength gradually decrease. The bond stress between the reinforcement and concrete shows a decreasing trend, whereas the maximum slip exhibits an increasing trend. Furthermore, a finite element model of the reinforced concrete pull-out specimen was established using ABAQUS software to simulate the bond performance under different freeze–thaw cycles. The comparison between experimental and simulated results validates the rationality of the finite element model. This study provides a reference for understanding the bond–slip behavior of tunnel lining reinforced concrete subjected to freeze–thaw environments in cold plateau regions.

## 1. Introduction

**Figure 1:**
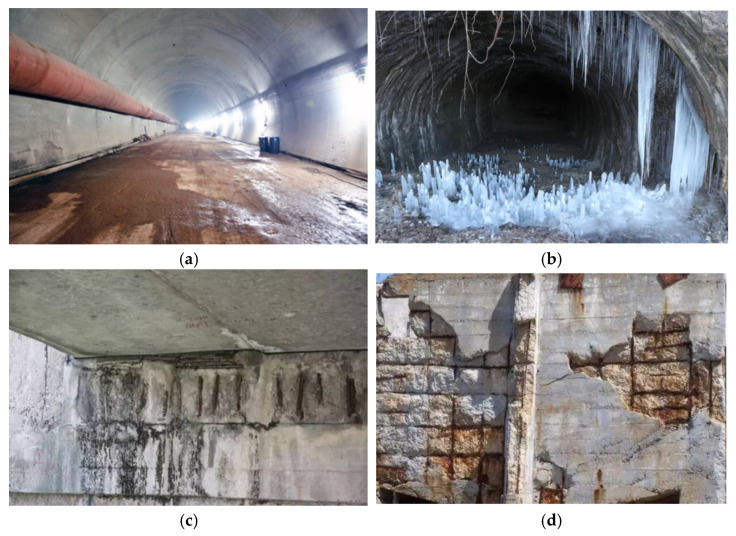
Field damage observed in concrete structures subjected to long-term freeze–thaw cycles and reinforcement corrosion: (**a**) pedestrian tunnel with visible surface deterioration; (**b**) frozen water melting and seepage inside a tunnel lining; (**c**) destructive spalling and exposure of corroded reinforced concrete structure; (**d**) structural cracking and scaling damage induced by freeze–thaw cycling.

The climate in plateau regions is characterized by a daily temperature difference typical of a cold temperate monsoon climate and plateau climate, with temperatures decreasing with increasing elevation. The region experiences low temperatures, significant daily temperature variations, and a low annual average temperature, leading to sharp temperature fluctuations. Consequently, the air density in this area is low, solar radiation is intense, and the duration of sunlight is long, resulting in a large annual temperature range and considerable day–night temperature differences [1]. In winter and summer, the maximum temperature difference exceeds 50 °C, and the maximum day–night temperature difference can reach 20 °C [2]. Due to the unique climate conditions characterized by significant temperature fluctuations, reinforced concrete structures, such as the lining at tunnel entrances, are subjected to freeze–thaw cycles year-round, leading to common forms of damage such as cracking, spalling, delamination, water leakage, frost heaving, and reinforcement corrosion [3], as illustrated in Figure 1.

The primary factor contributing to the damage of concrete in freeze–thaw environments is the formation of ice from water within its pore structure, leading to the generation of expansive stresses. As early as 1945, T.C. Powers [4] proposed the theory of hydrostatic pressure, which posits that, when water in the voids of concrete transitions from a liquid to a solid state (ice), localized hydrostatic pressure is generated. If this hydrostatic pressure exceeds the tensile strength of the concrete, it can result in internal structural failure. However, the hydrostatic pressure theory only accounts for some instances of damage caused by the volume expansion of liquids due to freeze–thaw cycles, and it does not explain damage occurring when some liquids do not undergo volume changes during freezing, highlighting certain limitations of the theory. Subsequently, T.C. Powers and Hwlmuth [5] introduced the theory of osmotic pressure, which focuses on the increase in the concentration of the residual solution after part of the moisture has turned to ice, leading to a significant osmotic pressure differential. If this osmotic pressure differential exceeds the strength limits of the concrete structure, it can also result in cracking. Together, these two theories reveal the potential threat posed by moisture to the integrity of concrete structures in freezing environments. To date, there has been extensive and in-depth academic exploration of the mechanisms of freeze–thaw damage in concrete, with various theoretical classifications. For instance, several existing theories include the moisture separation layer theory, expansive pressure theory, osmotic pressure theory, permeability coefficient theory, critical saturation value theory, and pore structure theory. In recent years, scholars have begun to focus on the multi-factor coupling effects of freeze–thaw cycles with chemical actions such as chloride penetration, sulfate attack, carbonation, and alkali–silica reactions on the degradation mechanisms of concrete [6].

The exploration of the bonding mechanism between reinforcement and concrete has progressed more slowly compared to other structural theories, initially relying on experimental methods and later advancing through theoretical derivation and numerical simulation. In 1951, Mains R.M. [7] innovatively embedded strain gauges on the surface of the reinforcement and determined the bonding stress distribution along the axis for smooth and deformed bars through pull-out tests. Mains R.M. was the first to clarify that bonding effectiveness is shaped by a combination of chemical bonding, frictional resistance, and mechanical anchorage. Subsequent scholars conducted extensive tests on bonding strength and derived new testing schemes, such as eccentricity diagrams, anchorage beam tests, and splice beam tests [8,9], based on the commonly used pull-out [10] and beam tests [11]. Additionally, with advancements in finite element software tools, many numerical models have been developed to further analyze testing conditions, primarily through contact analysis at RC interfaces [12] or by adding adhesive elements between RC [13]. Further research has also introduced new numerical models, such as fiber models [14] and concrete damage plasticity models [15].

In cold region environments, the repeated action of freeze–thaw cycles on reinforced concrete leads to the degradation of bonding between the concrete and reinforcement. In recent decades, numerous studies have focused on investigating the main factors affecting the bond strength between ordinary concrete and reinforcement under freeze–thaw conditions, including reinforcement properties, concrete properties, humidity, and temperature. Hanjari K.Z. [16] conducted experimental research on the effects of freeze–thaw cycles on the bonding performance between ordinary concrete and reinforcement. Through center pull-out tests, he found that increased freeze–thaw damage negatively impacts both bonding strength and initial stiffness, and also leads to an increase in the slip value corresponding to the maximum bonding stress. Xu S. [17] and colleagues explored the changes in interfacial bond strength between reinforcement and different grades of concrete under freeze–thaw action. Their experimental data indicated that, as the number of freeze–thaw cycles increased, the maximum bonding stress corresponding to slip values also increased for concrete of the same strength grade, while both bonding strength and stiffness decreased. Enhancing the concrete strength can improve bonding performance under freeze–thaw effects, and they developed a predictive model for the bond–slip relationship based on the experimental results. Additionally, Zhu F.’s team [18] conducted detailed experimental studies analyzing the effects of freeze–thaw cycles and reinforcement corrosion on the bonding performance between concrete and reinforcement. The results showed that, with an increasing number of freeze–thaw cycles, especially after multiple cycles, the bonding strength between corroded reinforcement and concrete significantly decreased, while slip performance noticeably increased. Moreover, a close correlation was observed between bonding strength, slip extent, and the number of freeze–thaw cycles.

Although existing studies have usefully explored the bond performance between ordinary concrete and steel reinforcement under freeze–thaw cycles and have established several bond–slip constitutive models, research on reinforced concrete (RC) structures of tunnel secondary linings in the complex climate conditions of plateau regions remains insufficient. In particular, most existing models are based on conventional concrete materials and lack systematic numerical simulation and validation that account for the mixed proportion characteristics of plateau lining concrete and the meso-scale damage mechanisms after freeze–thaw deterioration. Moreover, previous pull-out tests have mostly focused on the macroscopic degradation of bond strength, while the evolution of interface failure morphology between steel and concrete during freeze–thaw processes and its intrinsic correlation with the degradation of material mechanical properties have not been thoroughly explored.

To this end, this study takes the RC structure of the secondary lining of tunnels on the Qinghai–Tibet Plateau as the research object. Through systematic pull-out tests and ABAQUS (2024) finite element numerical simulations, this study focuses on revealing the evolution law of the bond–slip performance between lining concrete and steel reinforcement under different numbers of freeze–thaw cycles. Based on the experimental data, a numerical model applicable to the mechanical behavior of RC under freeze–thaw damage conditions is established. This study aims to deepen the understanding of the cooperative work performance between lining concrete and steel reinforcement under the influence of freeze–thaw cycles on plateau tunnels, thereby providing a theoretical basis for the durability design of RC tunnel structures in plateau regions.

## 2. Experimental Design

### 2.1. Raw Materials

**Figure 2:**
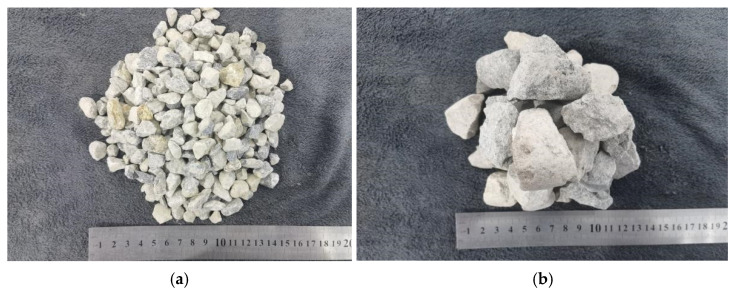
Aggregate selected for concrete mixture design: (**a**) coarse aggregate fraction with particle size 5–10 mm; (**b**) coarse aggregate fraction with particle size 10–20 mm.

In this experiment, the ordinary Portland cement P.O 42.5 was produced by Xizang Gaozheng Commercial Concrete Co., Ltd., Inside the Courtyard of Gaozheng Building Materials Co., Ltd., Jiamugou, Naqiong Town, Doilungdêqên District, Lhasa 850000, Tibet, China, with its chemical composition and basic performance indicators detailed in Table 1 and Table 2. The reinforcement selected was hot-rolled ribbed steel bars HRB335 from a metal materials company in Lhasa, with a diameter of 20 mm. The coarse aggregate was continuous-graded crushed stone provided by Xizang Gaozheng Commercial Concrete Co., Ltd., with sizes ranging from 5 to 20 mm, where the mass ratio of the 5–10 mm and 10–20 mm graded aggregates was 1:1.5. The fine aggregate consisted of medium sand from the banks of the Lhasa River, with a moisture content of 6% and a fineness modulus of 2.6 (as shown in Figure 2). Municipal drinking water from Lhasa was used for mixing and curing. The water-reducing agent was a polycarboxylic acid-based high-efficiency air-entraining water reducer produced by Lhasa Qili Building Materials Co., Ltd., No. 8 Guihua Road, Yangda Township Industrial Park, Doilungdêqên District, Lhasa 850000, Tibet, China, with a dosage of 2%. Limestone flour was supplied by Xizang Gaozheng Commercial Concrete Co., Ltd.

### 2.2. Specimen Preparation

#### 2.2.1. Concrete Mix Design

According to the requirements of GB 51081-2015 [19], the compressive strength of concrete under low-temperature conditions should not be lower than 40 MPa. Therefore, concrete with a design strength grade of C40 was used to ensure the general applicability of the test results. According to JGJ 55-2011 “Specification for Mix Proportion Design of Ordinary Concrete” [20], the mass per cubic meter of the constituent materials for the plateau tunnel lining concrete is presented in Table 3. As the objective of this study is to explore the evolution of bond–slip behavior between plateau lining concrete and reinforcement under freeze–thaw cycles and to characterize the degradation pattern of bond strength, the test specimens were designed using the same parameters as the actual plateau tunnel lining concrete.

#### 2.2.2. Fixture Production Required for the Test

**Figure 3:**
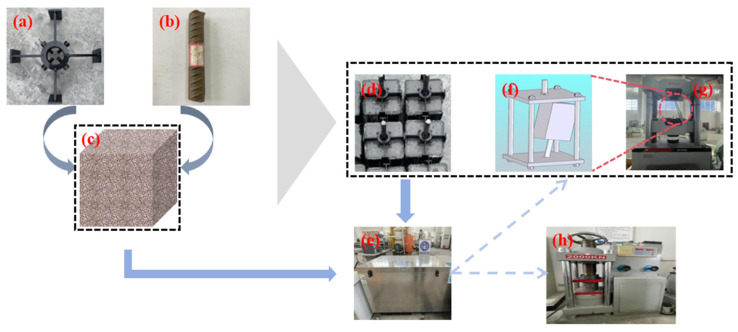
Experimental process illustrating specimen preparation and testing setup: (**a**) custom-designed fixture for specimen positioning; (**b**) steel reinforcement bars prior to casting; (**c**) freshly cast concrete specimens; (**d**) pull-out specimens after curing; (**e**) rapid freeze–thaw cycle testing machine; (**f**) drawing fixture mounted on the universal testing machine; (**g**) universal servo testing machine for pull-out tests; (**h**) microcomputer-controlled electro-hydraulic servo pressure testing machine for compressive strength measurement.

According to the design requirements of this experiment, the dimensions of the concrete specimens were 150 mm × 150 mm × 150 mm. To ensure that the reinforcement remained vertical to the concrete mold during the forming process, a fixture was designed, as shown in Figure 3a, to maintain the correct positioning of the reinforcement. The dimensions of this fixture matched those of the concrete specimen, with four threaded screws used to secure the reinforcement in place.

For the reinforced concrete specimens that met the experimental design requirements, pull-out tests were to be conducted immediately. To minimize experimental errors, a fixed clamp was designed and fabricated to secure the reinforced concrete specimens for the pull-out tests, taking into account the gripping distance of the reinforcement at both ends of the universal servo testing machine. The pull-out specimen fixture is shown in Figure 3f.

#### 2.2.3. Manufacture and Maintenance of Specimens

According to GBT 50082-2009 [21], hydrophobic release agents should be prohibited during the preparation of concrete specimens to ensure the accuracy of the tests. In addition, cubic compressive strength specimens measuring 100 mm × 100 mm × 100 mm should be prepared at the same age to comprehensively evaluate the properties of the concrete. During the specimen preparation process, the vibrating table and mixer used must comply with the relevant provisions of JG/T 245 [22] and JG 244 [23]. Furthermore, the preparation and curing of the specimens must adhere to GB/T 50081-2019, ensuring natural curing in a controlled indoor environment at a temperature of (20 ± 2) °C and relative humidity of (60% ± 5%) for 28 days to guarantee the scientific validity and effectiveness of the test results. The specimen preparation process for the pull-out tests, in accordance with the above standards for the long-term performance of concrete, is illustrated in Figure 3.

### 2.3. Test Scheme

The experimental methods in this study include rapid freeze–thaw cycling tests for concrete, cubic compressive strength tests, and bond strength tests. All specimens were prepared in accordance with the requirements of GB/T 50082-2009 [21] and the experiments were conducted following GB/T 50512-2012 [24]. The specific experimental procedures and standards are detailed as follows.

#### 2.3.1. Freeze–Thaw Cycle Test of Concrete

The freeze–thaw cycling tests for concrete are categorized into rapid freezing and slow freezing methods. This study utilized the TDR-III-type rapid freeze–thaw cycling testing machine, as shown in Figure 3e, employing the rapid freezing method for the experiments. Each set of 25 cycles constitutes a small cycle, with one freeze–thaw cycle completed every 4 h. The rapid freeze–thaw testing machine complies with the current industry standard JG/T 243 [25]. Temperature sensors were placed at the center of the temperature measuring specimen, in the antifreeze solution center inside the testing machine, and at both diagonal ends to monitor the temperature during the freeze–thaw cycles. It was ensured that the temperature difference among various points in the antifreeze solution within the test chamber remained ≤2 °C during operation.

In accordance with GBT 50082-2009, the reinforced concrete specimens with a side length of 150 mm were cured in a standard curing room for 28 days after molding. Upon completion of the curing period, the specimens were removed and the surface moisture was wiped with a dry towel before being swiftly placed into the rapid freeze–thaw cycling testing machine. Water was added to the rubber testing box containing the specimens to ensure that the water level was at least 5 mm above the top surface of the reinforced concrete specimens. The rapid freeze–thaw testing machine was then activated to conduct the experiments. Each time the specified number of freeze–thaw cycles was reached, the specimens were immediately removed, their surfaces cleaned of any residue, and were then promptly subjected to pull-out tests using a universal servo testing machine, with data recorded accordingly.

To investigate the effect of different numbers of freeze–thaw cycles on the ultimate bond–slip between reinforcing steel and concrete, hot-rolled ribbed steel bars with a diameter of 20 mm were uniformly used, and the embedded depth of the bars in the concrete was 75 mm. Five groups of specimens were subjected to 0 (control group), 50, 100, 150, and 200 freeze–thaw cycles, respectively. Three specimens were designed for each group, and the average value was taken as the final result.

#### 2.3.2. Test on Mechanical Properties of Concrete After Freeze–Thaw Cycles

Before testing the mechanical properties of the tunnel lining concrete after freeze–thaw cycling, the mass changes in the concrete cubic specimens were first measured at different freeze–thaw cycle counts. The mass loss rate of the concrete at varying freeze–thaw cycles was calculated according to Equation (1), and the average of the measurement results from each group of specimens was taken as the final value:

(1) $$∆W_{n}=\frac{W_{c}-W_{n}}{W_{0}}\times 100\%$$

where $W_{0}$ represents the initial mass of the specimen before freeze–thaw cycling (g), and $W_{n}$ denotes the mass of the specimen after *n* freeze–thaw cycles (g).

When evaluating the mass loss rate of the specimens, any values below zero in the experimental data should be treated as zero. If any extreme value among the three data points deviates from the median by more than 1%, that extreme value should be discarded, and the average of the remaining two should be used instead. If both the maximum and minimum values deviate from the median by more than 1%, the median should be taken as the final result.

In accordance with the requirements of GBT 50081-2019 [26], compressive and splitting strength tests were performed on concrete cubic specimens (100 mm × 100 mm × 100 mm) using a microcomputer-controlled electro-hydraulic servo pressure testing machine with a maximum load of 2000 kN, as shown in Figure 3h. The mechanical performance testing process is illustrated in Figure 3.

According to the freeze–thaw cycling test design requirements outlined in Table 4, for a total of five groups of different freeze–thaw cycling tests, each group required three compressive strength specimens and three splitting strength specimens, resulting in a total of six concrete cubic specimens with a side length of 100 mm. The specific experimental steps are as follows: (1) First, wipe the concrete specimens with severe surface mortar spalling to ensure a level state for testing. (2) Place the cleaned specimens horizontally in the center position of the pressure testing machine’s loading plate. (3) Start the pressure testing machine and apply a continuous and uniform load according to the testing standards. (4) Record the load value at the moment of failure.

#### 2.3.3. Test on Pull-Out Strength of Reinforced Concrete After Freeze–Thaw Cycles

In accordance with the requirements of GBT 50081-2019, pull-out tests on reinforced concrete were conducted using a universal servo testing machine, as shown in Figure 3g.

The freeze–thawed reinforced concrete specimens were inverted onto the pull-out fixture, with the rebar at one end embedded in the lower end of the universal servo testing machine, and securely fixed to the mold wall for loading purposes. The pull-out strength test for the concrete and rebar should follow these specific steps:

(1) The rebar must not be touched during the demolding process both before and after the specimen is formed, as well as upon reaching the designated testing age. The optimal time for demolding is within two days after forming, and care must be taken to remove the mold from the rebar.

(2) After reaching the specified curing period, the specimens should be removed from the standard curing environment and their surfaces cleaned. Additionally, the specimens must be inspected for any visible damage, such as missing concrete corners or abnormal rebar positions; testing should only proceed if no issues are found.

(3) The specimens should be inverted onto a steel plate with a central hole, ensuring that the concrete surface is level with the fixture’s steel plate, allowing the lower jaw of the pull-out testing machine to secure the rebar.

(4) During the setup of the testing apparatus, the stability of the micrometer gauge’s support frame should be ensured, and the gauge’s contact rod should be in contact with and perpendicular to the top of the rebar. Prior to loading, the uniformity and sensitivity of the contact rod should be carefully checked and calibrated. The initial reading of the micrometer gauge should be recorded before starting the universal servo tensile testing machine. The loading rate during the pull-out of the rebar should be maintained below 400 N/s. For loads in the range of 1 kN to 5 kN, the corresponding micrometer gauge readings should be recorded with each increment of load.

(5) The bond stress between the concrete and the rebar should be calculated according to the following Formulas (2) and (3):

(2) $$\tau =\frac{F}{A}$$

(3) A=πDL

where τ represents the bond stress of the rebar (MPa), with calculated results precise to 0.01 MPa; *F* is the maximum pull-out load of the rebar (N); *A* denotes the contact surface area between the concrete and the rebar (mm^2^); *D* is the diameter of the rebar (mm); and *L* is the embedded depth of the rebar (mm).

The bond strength test was designed to investigate the influence of the number of freeze–thaw cycles on the bond behavior of reinforced concrete. When using a ribbed rebar for testing, the arithmetic mean of the sliding deformation data from three specimens at each load level will be calculated and used as the x-coordinate for the relationship curve, while the corresponding load serves as the y-coordinate to plot the load versus sliding deformation curve. Subsequently, points corresponding to sliding deformations of 0.01 mm, 0.05 mm, and 0.10 mm will be identified on the curve to accurately read the corresponding load values.

## 3. Basic Properties of Reinforced Concrete Under Freeze–Thaw Damage

### 3.1. Effect of Freeze–Thaw Damage on Concrete Quality Change

**Figure 4:**
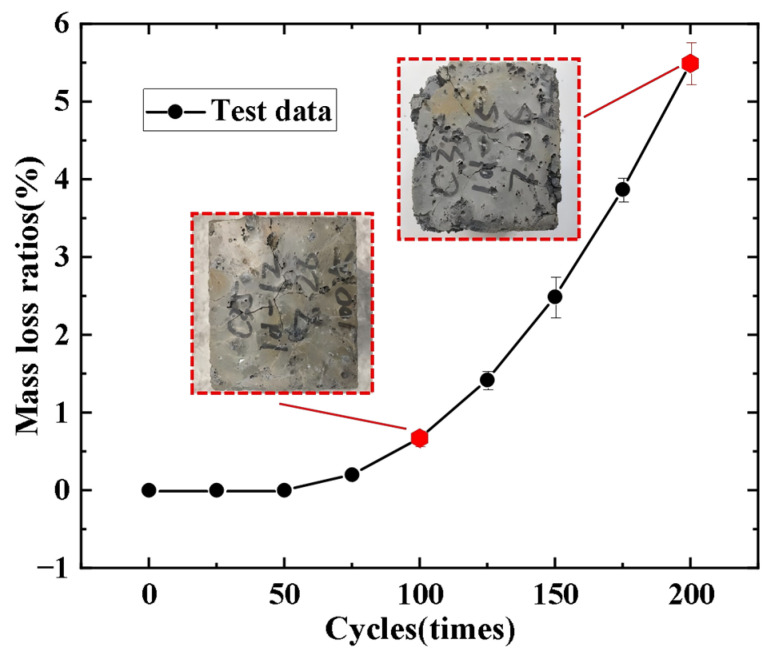
The mass loss rate of concrete changes with the number of freeze–thaw cycles.

This study first measures the initial mass of the specimens after curing is complete. Subsequently, after every 25 freeze–thaw cycles, the specimens are removed, their surfaces are dried with a cloth to remove moisture and debris, and their mass is recorded. Once the measurements are completed, the specimens are returned to the rapid freeze–thaw testing chamber for the next cycle. After undergoing varying numbers of freeze–thaw cycles, the concrete exhibits visible changes such as holes and cracks. Figure 4 illustrates the damage sustained by the concrete specimens after 100 and 200 freeze–thaw cycles.

The surface morphology of the concrete specimens reveals that, compared to the specimens immediately after curing, those subjected to 100 freeze–thaw cycles exhibit fine cracks and some small-diameter holes; however, no significant loss or erosion is evident at this stage. In contrast, the concrete specimens after 200 freeze–thaw cycles show numerous large-diameter holes and signs of ‘bulging’. There is a substantial overflow of mortar within the holes, and significant mortar spalling occurs at the edges, indicating severe freeze–thaw damage. The loss and spalling of the mortar on the concrete surface result in varying degrees of mass loss for the specimens. The change in mass loss rate of the concrete under different freeze–thaw cycles is illustrated in Figure 4. It is evident from the figure that, before reaching 100 cycles, the mass loss rate of the concrete specimens increases gradually; however, after 100 freeze–thaw cycles, the mass loss rate begins to rise rapidly, with a slight decrease noted at 25 cycles. After 200 freeze–thaw cycles, the mass loss rate reached 5% compared to the initial mass. This phenomenon occurs because, upon being placed in the rapid freeze–thaw testing machine, the ample water within the machine saturates the concrete specimen’s pores, resulting in the maximum mass. Following the freeze–thaw cycles, some free water within the pores transitions from liquid to solid and expands, which disrupts the bond between the coarse aggregate and the cementitious material in the concrete [27], leading to separation and the formation of varying-sized particulate debris, thus rapidly reducing the mass of the concrete. In the initial stages of the freeze–thaw cycles, the lower quantity and smaller diameter of pores result in a relatively minor volume change in the free water transitioning from liquid to solid, limiting the degree of mortar separation from the aggregates and resulting in a smaller mass loss rate. However, as the number of cycles increases, the loss of mortar leads to a significant increase in both the number and diameter of the pores. The degree of separation between the mortar and the coarse and fine aggregates increases, leading to the emergence of numerous holes on the surface of the specimens, resulting in substantial spalling of the mortar and exposing some coarse and fine aggregates, thus causing a dramatic decline in mass.

### 3.2. The Variation of Mechanical Properties of Concrete Under Freeze–Thaw Damage

This study is designed based on five gradients of freeze–thaw cycles (0, 50, 100, 150, and 200 cycles), with each group consisting of six specimens, including three for compressive strength testing and three for splitting tensile strength testing. After measuring the compressive strength and splitting tensile strength of the concrete specimens subjected to different freeze–thaw cycles, the loss rates of cubic compressive strength and splitting tensile strength are calculated using Equation (4), with the average values of the measurements from each group of specimens reported:

(4) $$∆f_{c}\left(n\right)=\frac{f_{c,0}-f_{c,n}}{f_{c,0}}\times 100\%$$

where $f_{c,0}$ represents the cubic compressive and splitting tensile strength before the freeze–thaw cycles (MPa) and ${\;f}_{c,n}$ denotes the cubic compressive and splitting tensile strength after *n* freeze–thaw cycles (MPa).

**Figure 5:**
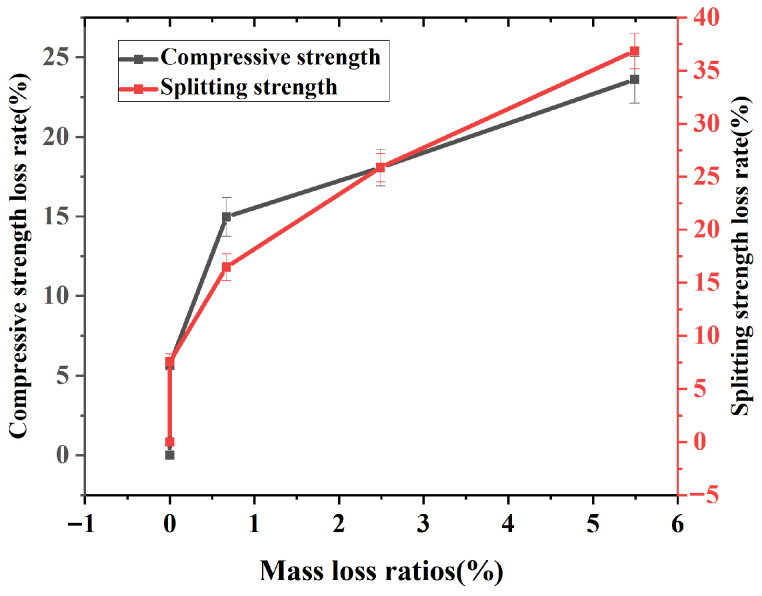
The variation of compressive strength and splitting strength loss rate with mass loss rate.

The relationship between the concrete strength loss rate and the mass loss rate is shown in Figure 5. From the figure, it is evident that the loss rates of both compressive strength and splitting tensile strength follow a similar trend as the mass loss rate increases. In the initial stage, the strength of the concrete continuously declines while the mass remains relatively stable. Subsequently, as the mass loss rate increases, the loss rates of both compressive and splitting tensile strengths increase almost linearly. This indicates that, with the increase in freeze–thaw cycle counts, the significant loss of concrete mortar leads to a gradual loosening of the structure, thereby reducing its mechanical properties.

## 4. Experimental Study on Bond Behavior of Reinforced Concrete Under Different Freeze–Thaw Cycles

### 4.1. Failure Mode of Reinforced Concrete Under Freeze–Thaw Damage

**Figure 6:**
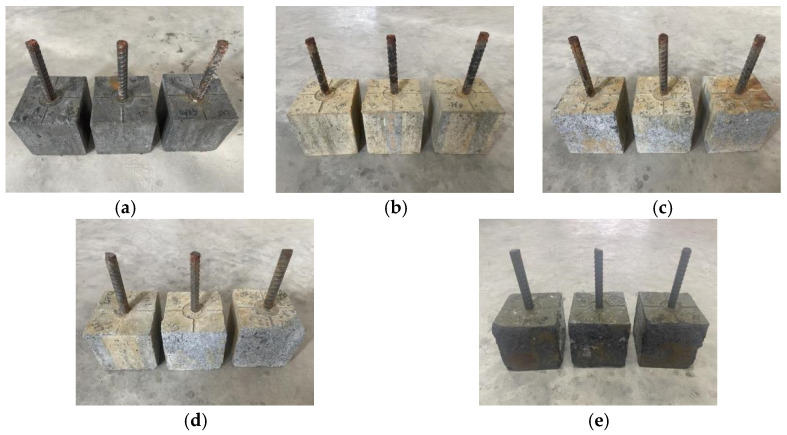
Surface damage morphology of reinforced concrete pull-out specimens subjected to varying numbers of rapid freeze–thaw cycles: (**a**) 0 cycles (reference undamaged state); (**b**) 50 cycles; (**c**) 100 cycles; (**d**) 150 cycles; (**e**) 200 cycles.

Figure 6 illustrates the changes in the surface morphology of the reinforced concrete specimens during the pull-out test as the number of freeze–thaw cycles increases. It is evident that, in the early stages of freeze–thaw cycles (0 and 50 cycles), the appearance of the reinforced concrete specimens does not show significant changes. However, as the number of freeze–thaw cycles reaches 100, the diameter of surface voids increases, leading to the formation of localized damage areas between the voids, with partial spalling of the surface mortar and rusting observed at the ends of the rebars. After 150 freeze–thaw cycles, the overall spalling of the specimen surface begins, with coarse aggregates becoming prominently exposed and local corner spalling occurring, accompanied by rusting on the exposed surfaces of the rebars. Following 200 freeze–thaw cycles, the extent of mortar loss further increases, resulting in more exposed coarse aggregates and a greater number of spalling areas on the concrete surface. Additionally, rust spots from the corroded rebars fall onto the concrete, causing the embedded segments of the rebars to gradually corrode.

### 4.2. Failure Mode of Reinforced Concrete Pull-Out Test

**Figure 7:**
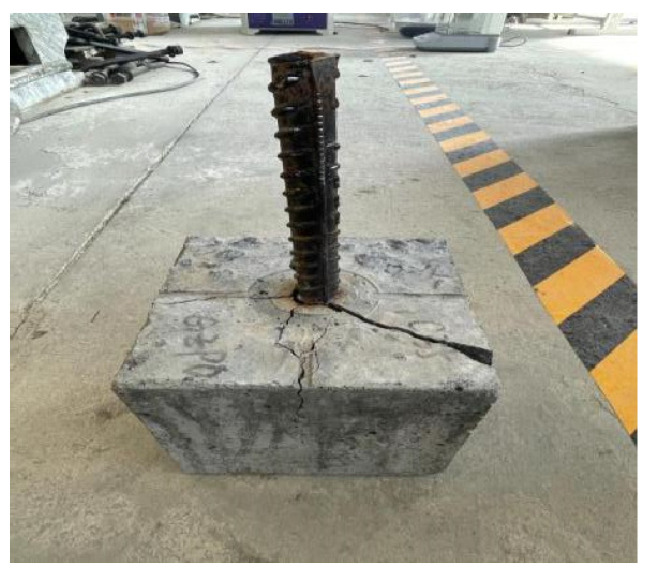
Splitting failure morphology of the pull-out specimen.

The pull-out failure modes of reinforced concrete can be categorized into three main types: pull-out failure, splitting failure, and yielding failure [28]. During the pull-out process, if no sound is emitted and no penetrating cracks appear on the concrete surface, it is classified as pull-out failure. If a crisp sound is heard and penetrating cracks develop on the concrete surface during the pull-out, it is identified as splitting failure. If the rebar does not get pulled out but instead reaches its yield strength prematurely, it is termed yielding failure. The failure morphology of some specimens after reinforcement pull-out is shown in Figure 7. Compared with the experimental results reported in the literature [29], it can be seen that all reinforced concrete specimens with hot-rolled ribbed bars of 20 mm diameter exhibited splitting failure, and the location of crack propagation due to splitting failure was approximately the same as the embedded depth of the reinforcement.

The bond strength between reinforced concrete is primarily categorized into three types: chemical bonding strength, mechanical interlocking strength, and frictional strength [30]. The vertical forces acting on the ribbed surface of the rebar ensure a firm bond with the concrete, resisting pull-out forces. Frictional strength and chemical bonding strength act against the direction of relative slip, and the interaction between the rebar and concrete relies on complex bonding mechanisms. Environmental factors affecting these bonding strengths lead to different failure modes. Chemical bonding strength initially tightly connects the rebar to the concrete, providing the necessary conditions for the action of other bonding strengths. When the chemical bonding strength fails first, the concrete at the bond interface with the rebar is crushed under mechanical interlocking strength, subsequently leading to rebar pull-out and detachment. If the chemical bonding strength is substantial, the concrete in that area will be torn at the high points of the rebar under mechanical interlocking strength, separating from the surrounding concrete, with the rebar remaining at its initial position after pull-out. Mechanical interlocking strength is primarily observed after rebar pull-out, manifested by the formation and development of cracks around the concrete. This occurs because the mechanical interlocking strength compresses the concrete within the grooves along its direction of action, causing cracks to initiate at the corners of the ribs and propagate outward. Frictional strength primarily arises during the relative movement between the rebar and concrete, acting in the opposite direction to the pull-out of the rebar.

The bonding performance at the reinforced concrete interface is maintained through the combined action of chemical bonding, mechanical interlocking, and friction. Before sliding occurs in the reinforced concrete, chemical bonding and mechanical interlocking dominate. At this stage, the ribbed rebar induces diagonal loads, promoting the formation and gradual expansion of cracks within the concrete. As the load continues to increase, cracks develop rapidly, ultimately leading to initial cracking. Once cracks penetrate and extend to a specific threshold, the chemical bonding and mechanical interlocking strengths at the interface reach their limits, and the rebar begins to slide relative to the concrete. Although the load is not zero at this point, frictional strength remains active; however, as sliding continues, the contact area decreases, leading to a gradual reduction in frictional strength until the rebar is fully extracted and the load drops to zero [31].

### 4.3. Analysis of Test Results

**Figure 8:**
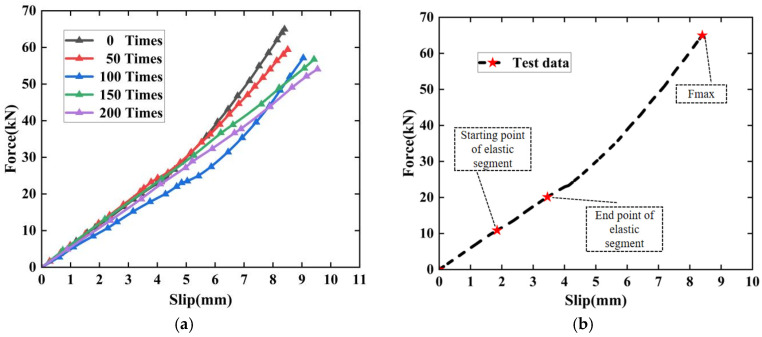
Bond–slip behavior of concrete specimens under different freeze–thaw conditions: (**a**) bond–slip curves after exposure to varying numbers of freeze–thaw cycles (0, 50, 100, 150, and 200 cycles); (**b**) schematic illustration of characteristic points on a typical bond–slip curve.

This study investigates the effects of different freeze–thaw cycles on the bond–slip characteristics between concrete and rebar by testing the average bond strength and slip values of reinforced concrete specimens. The bond stress values and maximum slip values at failure were recorded for specimens subjected to five different gradients of freeze–thaw cycles (0, 50, 100, 150, and 200) under the same conditions of rebar diameter and embedment depth. By adhering to bond strength specifications, the corresponding bond stress values for each slip value were determined, allowing for the calculation of the average bond stress for the reinforced concrete as a reference value to analyze the impact of varying freeze–thaw cycles on the bond stress between rebar and concrete. Figure 8 presents the continuous curves of bond strength and slip values for concrete specimens under different conditions (as shown in Figure 8).

**Figure 9:**
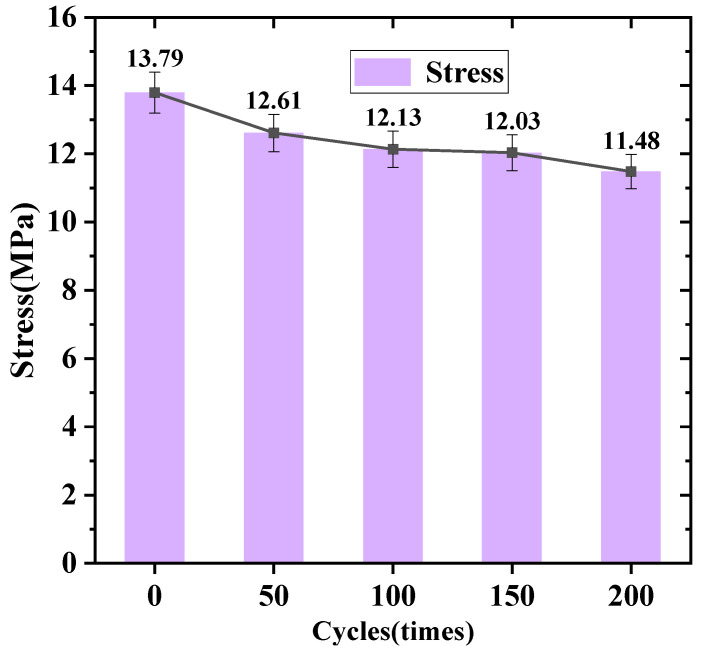
Variation of bond stress in pull-out specimens after exposure to different numbers of freeze–thaw cycles (0, 50, 100, 150, and 200 cycles).

The data were organized and analyzed to illustrate the trend of average bond stress as the number of freeze–thaw cycles increased, as shown in Figure 9. The results indicate that, for reinforced concrete pull-out specimens with a standard rebar embedment depth of 75 mm and a rebar diameter of 20 mm, the average bond strength decreases with an increasing number of freeze–thaw cycles. Specifically, after 200 freeze–thaw cycles, the average bond strength decreased by 1.7 MPa. Compared to the bond strength of concrete without freeze–thaw treatment, the bond strength decreased by 12.3% in this study. In the related study by Milad Shakiba et al. [32], after 200 freeze–thaw cycles, the bond strength of normal concrete decreased by 12%, which is generally consistent with the decrease observed in the plateau lining concrete of this study. For specimens cast with self-compacting concrete and fiber-reinforced concrete, the bond strengths decreased by 3% and 9%, respectively. Under the same treatment conditions, the corresponding values for GFRP-reinforced concrete beams were 1%, 1%, and 16%, respectively. Therefore, in the subsequent construction of tunnel lining concrete in plateau regions, fiber reinforcement of concrete can be considered to mitigate the effects of freeze–thaw damage, thereby extending its service life.

**Figure 10:**
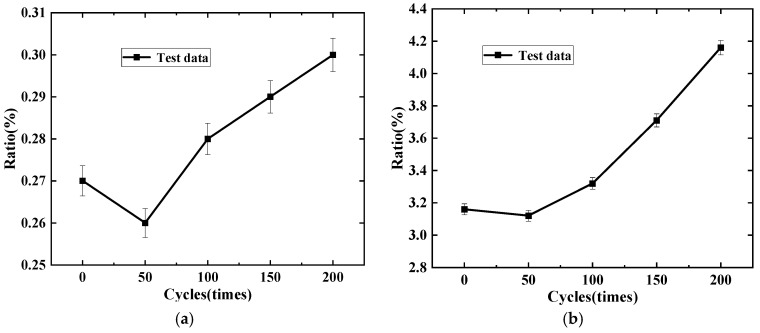
Relationship between normalized bond stress and freeze–thaw cycles: (**a**) the ratio of bond stress to compressive strength freeze–thaw cycles; (**b**) the ratio of bond stress to splitting tensile strength freeze–thaw cycles.

From the previous analysis, it is evident that the bond stress between the rebar and concrete is related to the concrete strength, both of which decrease with an increasing number of freeze–thaw cycles. Figure 10 establishes the relationship between bond stress values and the ratio of concrete strength to freeze–thaw cycles.

From Figure 10, it is evident that the bond stress values relative to concrete strength decrease slightly after 50 freeze–thaw cycles compared to 0 cycles. However, as the number of freeze–thaw cycles increases, the ratio of bond stress to concrete strength gradually increases, indicating that the impact of freeze–thaw cycles on the material’s inherent strength is more significant. The bond strength between rebar and concrete primarily relies on chemical adhesion and frictional forces as freeze–thaw cycles accumulate.

The average bond stress loss rate in reinforced concrete increases with the number of freeze–thaw cycles, reaching 17% after 200 cycles. The primary reasons for the impact of freeze–thaw cycles on the bond performance between rebar and concrete include changes in physical properties and internal pore structure. Physically, as the number of freeze–thaw cycles increases, both the compressive and splitting strengths of concrete gradually decline. During loading, the tensile yield strength of the rebar greatly exceeds the compressive and splitting strengths of the concrete. Consequently, when the limit bond stress is reached, the first failure occurs at the interface between the rebar and concrete, leading to reduced bond stress. Regarding internal pore changes, freeze–thaw cycles cause internal voids and cracks to connect and expand, further weakening the bond effectiveness between the concrete and rebar, which exacerbates the degradation of bond stress.

**Figure 11:**
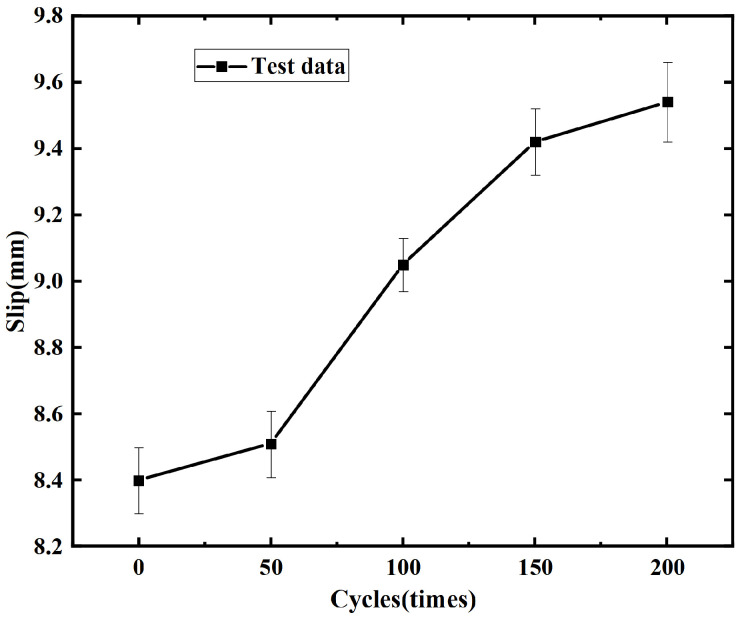
The change in the maximum slip value of different freeze–thaw cycles.

The maximum slip value of the rebar, corresponding to the ultimate bond strength of the reinforced concrete, has been analyzed and illustrated in Figure 11. It is clear that, under the same conditions of 75 mm rebar embedment depth and 20 mm diameter, the maximum slip value increases with the number of freeze–thaw cycles, with an increase of 1.1 mm after 200 cycles.

**Figure 12:**
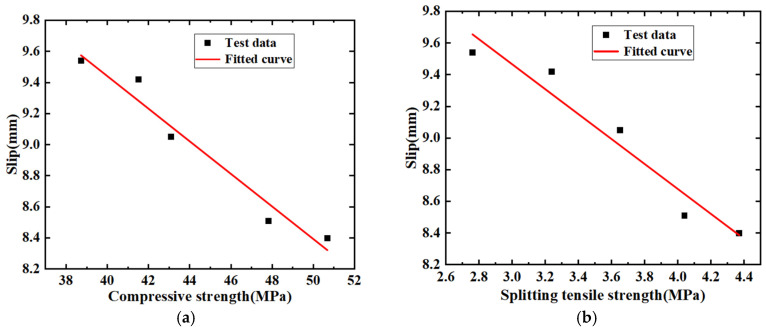
The relationship between the maximum slip value and the strength of concrete: (**a**) maximum slip value–compressive strength; (**b**) maximum slip value–splitting tensile strength.

From the figure, it can be observed that the growth rate of the maximum slip of the rebar increases with the number of freeze–thaw cycles. After 200 freeze–thaw cycles, the growth rate of the maximum slip reaches 13%. This phenomenon can be attributed to the increased formation of cracks at the interface between the rebar and concrete as the number of freeze–thaw cycles increases. Consequently, more water can flow into these cracks. Following freeze–thaw cycles, the volume of these voids increases, leading to the loosening of the concrete surrounding the rebar, which reduces the resistance to slip during loading, thereby resulting in a gradual increase in slip. Considering that the change in concrete strength is a key reason for the increase in slip values with the number of freeze–thaw cycles, a fitting relationship between the maximum slip of the rebar and the compressive and splitting strengths of concrete has been established, as shown in Figure 12 and Equations (5) and (6).

In Equations (5) and (6), *x* represents the slip value, while $\sigma_{p}$ and $\sigma_{s}$ denote the compressive and splitting strengths of the concrete, respectively. The parameter *n* equals 4, and the correlation coefficients *R* are 0.973 and 0.961. Given a significance level α = 0.005, the critical correlation coefficient $R_{0.005}$ = 0.942 < R.

The data and the fitting relationship indicate a clear linear relationship between the rebar slip values and concrete strength. The degradation of concrete strength due to loosening from freeze–thaw cycles significantly increases the maximum slip of the rebar embedded in the concrete.

(5) $$x=-1.105\times \sigma_{p}+13.634$$

(6) $$x=-0.788\times \sigma_{s}+11.830$$

## 5. Finite Element Simulation of Bond–Slip of Reinforced Concrete Under Freeze–Thaw Damage

In this study, the ABAQUS finite element analysis software was utilized to establish a bond–slip finite element model of reinforced concrete under freeze–thaw damage. Based on experimental data and modifications to the Petersen bond–slip constitutive relationship, the model was developed to analyze the effects of different freeze–thaw cycle counts on the bond–slip relationship between rebar and concrete. The bond–slip data extracted from the simulation results were thoroughly analyzed, and a comparison was made between the simulated values and experimental results. This comparison validated the effectiveness and applicability of the bond–slip model for reinforced concrete under freeze–thaw damage conditions.

### 5.1. Finite Element Model Selection

**Figure 13:**
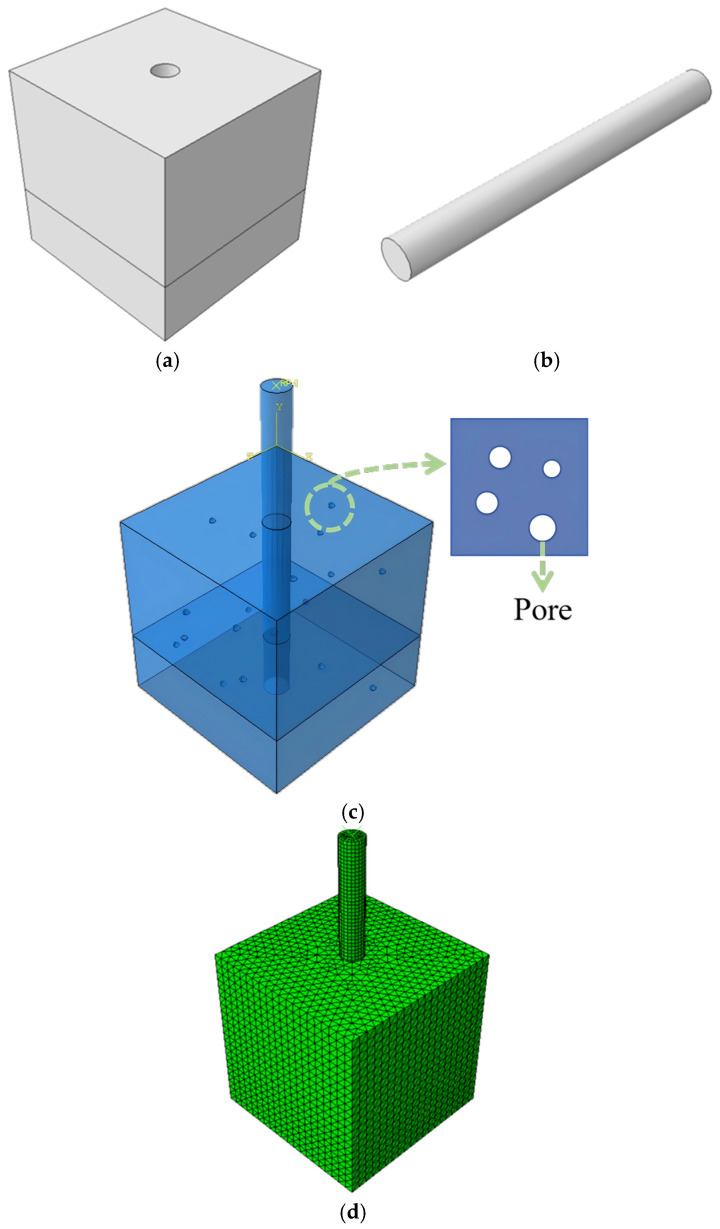
Finite element model of the reinforced concrete pull-out specimen: (**a**) concrete block component modeled; (**b**) reinforcing steel bar component modeled; (**c**) assembled model showing the embedded reinforcement; (**d**) mesh discretization of the complete pull-out model.

The finite element model dimensions are set to 150 mm × 150 mm × 150 mm, using concrete with a design strength grade of C40. The rebar diameter and embedment depth are specified according to the experimental design, utilizing a 20 mm diameter rebar with a 75 mm embedment depth. The established model is illustrated in Figure 13, and the unit meshing of the reinforced concrete allows for the simulation of the bond stress between the rebar and concrete.

### 5.2. Constitutive Model of Steel and Concrete Materials

#### 5.2.1. Constitutive Model of Steel Material

In establishing the finite element model for the rebar, this study utilizes the experimental data values for the rebar’s elastic modulus and tensile yield strength, with a Poisson’s ratio set at 0.30. Based on the stress–displacement curve obtained from experiments, a piecewise linear constitutive model is employed for the rebar.

#### 5.2.2. Constitutive Model of Concrete Material

Concrete, as an engineering material, exhibits complex mechanical behavior that necessitates precise constitutive models to ensure analytical accuracy. In ABAQUS, the numerical representation of concrete employs a method based on the continuum medium hypothesis rather than simulating macro crack propagation. Specifically, for concrete structures under low confining pressure conditions, this study adopts the Concrete Damaged Plasticity (CDP) model [33]. This model effectively captures the nonlinear behavior of concrete when subjected to multi-axial loading, particularly at maximum uniaxial stress levels up to four to five times its capacity. The model distinguishes between different damage mechanisms and response characteristics under compression and tension, providing a reliable theoretical foundation for simulating concrete structures under complex stress paths.

In this study, the fundamental physical parameters of concrete in the finite element analysis model were determined based on experimental data, with a density of 2500 kg/m^3^ and a Poisson’s ratio of 0.30. Furthermore, the concrete damage plasticity (CDP) model was adopted to simulate the mechanical behavior of concrete, which requires detailed definitions of parameters related to plasticity and damage. The parameters for the plasticity and damage parts of the model were adjusted according to the existing literature and the default recommendations of the software. Based on this, the dilation angle was set to 30°. In addition, the eccentricity was set to 0.1, the K value to 0.6667, and the ratio f_B0_/f_C0_ to 1.16. These parameters collectively define the failure behavior of the material under loading.

In the damage model section, a damage index and a stiffness recovery coefficient are introduced to detail the damage behavior of concrete during tensile and compressive processes. The damage index primarily describes the reduction in material stiffness with non-elastic strain, while the stiffness recovery coefficient simulates changes in stiffness under reverse loading. Since this research focuses on monotonic loading, the application of the damage model under cyclic loading is not addressed. The parameters for concrete’s behavior under compression and tension in the CDP model are calculated as shown in the following equations [34]:

(7) $$\partial_{c}=\;(1\;-\;d_{c})E_{0}(\varepsilon_{c}-\;\varepsilon_{c}^{pl})$$

(8) $$\varepsilon_{0c}^{el}=\partial_{c}-E_{0}$$

(9) $$\varepsilon_{c}^{in}=\varepsilon_{c}-\varepsilon_{0c}^{el}$$

(10) $$\varepsilon_{c}^{pl}=b_{c}\varepsilon_{c}^{in}$$

(11) $$d_{c}=1-\frac{\partial_{c}E_{0}^{-1}}{\varepsilon_{c}^{pl}(1/b_{c}-1)+\partial_{c}E_{0}^{-1}}$$

where $\varepsilon_{c}$, $\varepsilon_{0c}^{el}$, $\varepsilon_{c}^{in}$ and $\varepsilon_{c}^{pl}$ represent the compressive strain, elastic strain, inelastic strain, and plastic strain of concrete, respectively. The compressive stress $\partial_{c}$ is defined as $\partial_{c}$ ≤ 0.4$f_{c}$, indicating that concrete is in the elastic stage under compression [35]. $E_{0}$ is the elastic modulus of concrete, $b_{c}$ is the ratio coefficient between inelastic strain and plastic strain, taken as 0.7 [36], and $d_{c}$ is the compressive damage coefficient of concrete:

(12) $$\partial_{t}=(1\;-\;d_{t})E_{0}{(\varepsilon }_{t}-\varepsilon_{t}^{pl})$$

(13) $$\varepsilon_{0t}^{el}=\partial_{t}/E_{0}$$

(14) $$\varepsilon_{t}^{ck}=\varepsilon_{t}-\varepsilon_{0t}^{el}$$

(15) $$\varepsilon_{t}^{pl}=b_{t}-\varepsilon_{t}^{ck}$$

(16) $$d_{t}=1-\frac{\partial_{t}E_{0}^{-1}}{\varepsilon_{t}^{pl}(1/b_{t}-1)+\partial_{t}E_{0}^{-1}}$$

where $\varepsilon_{t}$, $\varepsilon_{0t}^{el}$, $\varepsilon_{t}^{ck}$ and $\varepsilon_{t}^{pl}$ represent the tensile strain, elastic strain, cracking strain, and plastic strain of concrete, respectively. The tensile stress $\partial_{t}$ is defined as $\partial_{t}$ ≤ $f_{t}$, indicating that concrete is in the elastic stage under tension [36]. $E_{0}$ is the elastic modulus of concrete, $b_{t}$ is the ratio coefficient between cracking strain and plastic strain, taken as 0.1 [37], and $d_{t}$ is the tensile damage coefficient of concrete.

### 5.3. Finite Element Simulation Results

**Figure 14:**
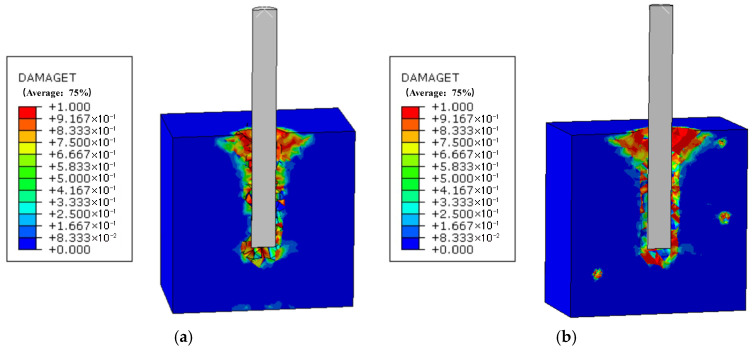

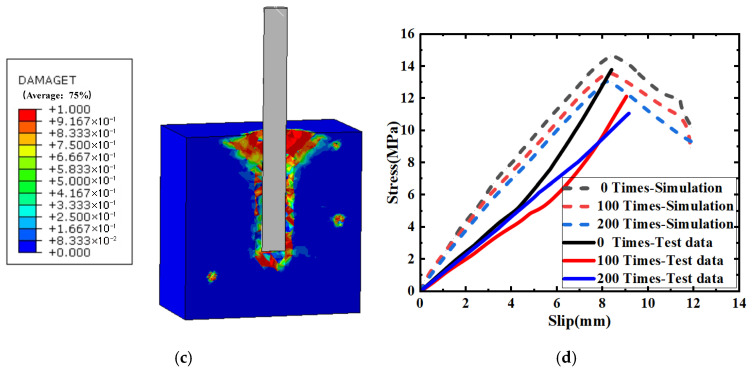
Finite element analysis results of reinforced concrete pull-out specimens under freeze–thaw exposure: (**a**) 0 freeze–thaw cycles; (**b**) 100 freeze–thaw cycles; (**c**) 200 freeze–thaw cycles; (**d**) numerical simulation of bond–slip curve corresponding to the different freeze–thaw cycles.

Using the number of freeze–thaw cycles of reinforced concrete as a variable, simulations were conducted on pull-out specimens with a steel diameter of 20 mm and an embedment depth of 75 mm, specifically at 0, 100, and 200 freeze–thaw cycles. The damage factor cloud maps for peak load under different freeze–thaw cycles are shown in Figure 14. From the figure, it is evident that damage manifests as cracks developing in the same direction as the pull-out of the reinforcement, indicating that, for 20 mm diameter steel bars under varying freeze–thaw cycles, the failure primarily exhibits splitting failure. During the pull-out process, concrete damage is primarily concentrated around the steel bars. As the number of cycles increases, the damage at the base of the concrete becomes progressively more severe. The pull-out damage distribution for specimens subjected to 200 freeze–thaw cycles is noticeably denser compared to those subjected to 0 freeze–thaw cycles, and the area of pull-out damage increases with the number of freeze–thaw cycles, which is consistent with the experimental results.

Through ABAQUS finite element numerical simulation analysis, the relationship curves of bond stress versus slip distance for reinforced concrete specimens subjected to 0, 100, and 200 freeze–thaw cycles were plotted, as shown in Figure 14d. The results indicate that, as slip distance increases, the trend of bond stress under different freeze–thaw cycles is similar. The bond stress initially increases linearly with the slip distance. Upon reaching the ultimate bond stress, the bond stress gradually decreases until failure occurs. This demonstrates that the trend of the bond–slip curve remains unaffected by varying freeze–thaw cycles; however, the maximum bond stress of the reinforced concrete decreases with an increasing number of freeze–thaw cycles, consistent with the experimental findings.

By comparing the experimental data on bond stress under different freeze–thaw cycles with the finite element simulation results, as shown in Table 5, it is evident that the finite element simulation results closely align with the experimental results. The error values confirm the accuracy of the established finite element model for the pull-out specimens of reinforced concrete.

## 6. Conclusions

In this study, the fundamental mechanical properties of tunnel lining concrete and its bond performance with steel reinforcement bars subjected to freeze–thaw cycles were investigated using rapid freeze–thaw tests and center pull-out tests. Numerical simulations of the bond performance after freeze–thaw damage were also performed using the ABAQUS finite element software. Based on the experimental results and within the limitations of the present test conditions, the following preliminary conclusions can be drawn.

(1) Within the range from 0 to 200 freeze–thaw cycles considered in this study, the mass loss rate of the lining concrete specimens first decreased slightly and then increased, indicating an initial minor mass gain followed by a loss. Both the cubic compressive strength and splitting tensile strength of the concrete decreased with an increasing number of freeze–thaw cycles, with the splitting tensile strength exhibiting a more pronounced decreasing trend than the compressive strength. In contrast, no significant change in the tensile strength of the steel bars was observed after different numbers of freeze–thaw cycles.

(2) As the number of freeze–thaw cycles increased, the average bond stress between the lining concrete and steel bars decreased, while the maximum slip corresponding to the ultimate bond stress increased. Regression analysis showed that, under the present test conditions, the bond stress decreased with decreasing compressive strength and splitting tensile strength of the concrete, whereas the maximum slip increased with decreasing values of both strength parameters. It should be noted that these fitting relationships were derived from a limited sample size and did not account for other potential influencing factors, such as the degree of steel corrosion and the distribution of internal pores in the concrete. Therefore, further validation is required.

(3) The numerical pull-out model of reinforced concrete established using ABAQUS yielded simulated bond stresses that were in good agreement with the experimental results under different numbers of freeze–thaw cycles, with a maximum error of less than 15%. This indicates that the numerical model is reasonably applicable under the specific conditions of this study. However, because the simulations were performed only for specific specimen dimensions, steel bar diameters, and embedment depths, the generalizability of the model to other conditions requires additional verification.

In summary, within the scope of this study, the number of freeze–thaw cycles significantly affected the bond performance between steel bars and concrete. The above results preliminarily reveal the degradation pattern of the bond performance between lining concrete and steel bars under freeze–thaw damage. However, the range of test conditions is relatively narrow (only the influence of freeze–thaw cycles is discussed). Future research should expand the range of freeze–thaw cycles, and systematically study the influence of steel bar diameter, buried depth, concrete mix ratio, concrete cover thickness, stirrup configuration and other parameters, in order to provide a more reliable basis for the durability design of reinforced concrete tunnel lining in plateau areas.

## Figures and Tables

**Table 1:** Chemical composition of cement/%.

SiO_2_	CaO	Fe_2_O_3_	Al_2_O_3_	Na_2_O	K_2_O	MgO	SO_3_	f-CaO	LOI
20.35	62.31	3.32	5.43	0.28	0.78	2.93	2.17	1.38	1.50

**Table 2:** Physical and mechanical properties of cement.

Standard Consistency/%	Cement Soundness	Setting Time/min		Flexural Strength/MPa		Compressive Strength /MPa	
		Initial Set	Initial Set	3 d	28 d	3 d	28 d
27%	Qualification	121	162	5.7	8.5	34	58.2

**Table 3:** Mix proportion of tunnel lining concrete.

Specimen Number	The Material Used Per m^3^ Concrete/kg·m^−3^						W/B
	Cement	Limestone Flour	Coarse Aggregate	Fine Aggregate	Total Water	Water Reducing/%	
C40	350	50	1043	250	170	2	0.379

**Table 4:** Specimen number of different freeze-thaw cycles.

Specimen Number	Diameter of Rebar (mm)	Types of Steel Bars	Reinforcement Embedded Depth (mm)	Number of Freeze-Thaw Cycles
D20-0-1	20	HRB335	75	0
D20-0-2	20	HRB335	75	0
D20-0-3	20	HRB335	75	0
D20-50-1	20	HRB335	75	50
D20-50-2	20	HRB335	75	50
D20-50-3	20	HRB335	75	50
D20-100-1	20	HRB335	75	100
D20-100-2	20	HRB335	75	100
D20-100-3	20	HRB335	75	100
D20-150-1	20	HRB335	75	150
D20-150-2	20	HRB335	75	150
D20-150-3	20	HRB335	75	150
D20-200-1	20	HPB335	75	200
D20-200-2	20	HPB335	75	200
D20-200-3	20	HRB335	75	200

**Table 5:** Comparison of simulation and test results of different freeze–thaw cycles.

Specimen Number	Bond Stress/MPa		
	Simulation	Test	Error/%
D20-0-75-1	14.69	13.79	6.5
D20-100-75-2	13.60	12.13	12.1
D20-200-75-3	13.11	11.48	14.2

## Data Availability

The original contributions presented in this study are included in the article. Further inquiries can be directed to the corresponding authors.
